# Tebentafusp induces systemic immune activation, but tumor immune remodeling remains incompletely validated: a narrative review in metastatic uveal melanoma

**DOI:** 10.3389/fimmu.2026.1934256

**Published:** 2026-10-12

**Authors:** Francesca Rifaldi, Filippo De Braud, Michele Del Vecchio, Lorenza Di Guardo

**Affiliations:** 1 Department of Internal Medicine and Medical Therapy, University of Pavia, Pavia, Italy; 2 Department of Oncology, Comprehensive Cancer Center, IRCCS Policlinico San Matteo Foundation, Pavia, Italy; 3 Department of Medical Oncology, Fondazione IRCCS Istituto Nazionale dei Tumori, Milan, Italy; 4 Melanoma Medical Oncology Unit, Department of Medical Oncology, Fondazione IRCCS Istituto Nazionale dei Tumori, Milan, Italy

**Keywords:** immune biomarkers, metastatic uveal melanoma, narrative review, PRISMA, T cell engager, TCR-CD3 bispecific, tebentafusp, tumor microenvironment

## Abstract

**Background:**

Tebentafusp improves survival in HLA-A*02:01-positive metastatic uveal melanoma, yet the human evidence linking early systemic immune activation to remodeling of metastatic tumors is dispersed across overlapping translational reports.

**Methods:**

We performed a PRISMA-style, search-informed narrative review and descriptive evidence mapping of human reports evaluating baseline or on-treatment immune measurements in tebentafusp-treated uveal melanoma. PubMed/MEDLINE, Embase, and CENTRAL were searched for reports published from January 2015 to 9 December 2025, with an updated search on 9 June 2026. Reports were linked to their underlying trial or cohort before synthesis.

**Results:**

Overall, 286 records were screened, 21 full-text reports or complete abstracts were assessed, and 10 reports were retained. These represented three principal evidence units rather than 10 independent studies: NCT02570308, NCT03070392, and an independent serial skin-biopsy cohort. Blood-based analyses consistently showed early cytokine induction and transient lymphocyte changes after tebentafusp dosing. Paired metastatic-tissue analyses reported increased CD3 and CD8 cell density and interferon-response programs by day 16, but these observations derived predominantly from overlapping analyses of NCT02570308. Baseline and on-treatment immune features were associated with survival in exploratory analyses. Skin biopsies provided independent evidence of on-target cutaneous pharmacodynamics but only indirect evidence regarding metastatic tumor remodeling.

**Conclusion:**

Tebentafusp is associated with consistently observed early systemic immune activation across overlapping analyses, but treatment-associated metastatic tumor immune remodeling remains incompletely validated. The available evidence supports temporal coherence rather than a proven causal systemic-to-tumor pathway. Future studies should use prospective paired blood and liver sampling, harmonized time points, prespecified immune endpoints, and mediation analyses linking immune changes to clinical outcomes.

## 1 Introduction

Metastatic uveal melanoma is biologically distinct from cutaneous melanoma and has a marked hepatic tropism. Its low tumor mutational burden and immunoregulatory metastatic microenvironment contribute to the limited activity of conventional immune checkpoint inhibition. Tebentafusp is an affinity-enhanced soluble T cell receptor fused to an anti-CD3 effector domain that recognizes a gp100 peptide presented by HLA-A*02:01 and redirects polyclonal T cells toward gp100-expressing cells. In the randomized phase 3 trial, tebentafusp improved overall survival despite a comparatively modest objective response rate, motivating investigation of pharmacodynamic and molecular correlates (1).

Human translational studies have reported rapid cytokine release, transient changes in circulating lymphocytes, increased T cell density in paired tumor biopsies, interferon-associated gene expression, and macrophage and B cell changes. These observations are frequently cited together as evidence that tebentafusp converts an immune-excluded tumor into an inflamed microenvironment. However, the literature contains overlapping analyses, preliminary conference reports, different biological compartments, and non-equivalent endpoints. Counting each publication as an independent study can therefore overstate replication.

The purpose of this review was not simply to catalogue known pharmacodynamic effects. Instead, we asked whether the available human evidence supports a temporally ordered systemic-to-tumor immune remodeling model: (1) early systemic activation, (2) trafficking, redistribution, or altered compartmental abundance of effector cells, (3) immune remodeling within metastatic tissue, and (4) association of these changes with clinical outcome. We additionally distinguished direct evidence from blood or metastatic tissue from indirect evidence derived from skin.

## 2 Search-informed review approach

### 2.1 Protocol and review position

The protocol was submitted to PROSPERO on 23 January 2026 and registered on 27 January 2026 (CRD420261292110). Because the original electronic searches had already been conducted on 9 December 2025, the registration was not fully prospective. The latest registered version (version 1.2) was published on 19 May 2026. The project was originally designed as a systematic review; however, because the available literature consisted mainly of overlapping exploratory translational reports and because the planned independent checks of screening, extraction, and appraisal were not completed, the manuscript was repositioned as a narrative descriptive review informed by systematic searching. A PRISMA-style flow diagram is provided for transparency (2), and the descriptive grouping was informed by SWiM principles where applicable (3), but the article should not be interpreted as a fully compliant formal systematic review with duplicate screening and domain-level risk-of-bias grading.

### 2.2 Information sources and search strategy

PubMed/MEDLINE, Embase, and the Cochrane Library (CENTRAL) were searched on 9 December 2025 for human studies published from January 2015 through the search date. Eligibility was restricted to reports published in English; a language limiter was not included in the electronic search strings. Search concepts combined uveal or ocular melanoma with tebentafusp, IMCgp100, ImmTAC, gp100-directed T cell receptor bispecifics, and immune or biomarker terms. Reference lists of eligible reports were examined for additional publications and conference reports. The complete database-specific strategies as originally executed are reported in **Supplementary Table S1**. When the strategies were rerun on 9 June 2026, they retrieved 45 records from PubMed/MEDLINE, 251 from Embase, and 29 from CENTRAL; applying an English-language filter reduced the PubMed and Embase results to 39 and 219, respectively.

### 2.3 Eligibility criteria

Eligible populations were adults with advanced or metastatic uveal melanoma treated with tebentafusp. Mixed melanoma populations were eligible only when uveal-melanoma results were separable. Eligibility was applied at the outcome level rather than automatically to an entire report: a report was retained only for outcomes that met the final review scope, and non-eligible outcomes within the same report were not synthesized as formal evidence. The final formal synthesis required tebentafusp-attributable immune pharmacodynamic outcomes that included cellular immune profiling in blood or metastatic tissue, or cytokine, chemokine, immune-related gene-expression, or immune-architecture findings reported within the same eligible cellular/tissue-immunology report or evidence unit. Stand-alone cytokine-only, chemokine-only, ctDNA-only, or clinical-efficacy reports were not retained in the formal synthesis when they lacked separable cellular immune-profiling outcomes. Serial skin-biopsy studies were retained as an explicitly labeled scope deviation and analyzed as an indirect pharmacodynamic model. Preclinical-only findings, including experimental macrophage models, were not treated as patient-sample evidence. Clinical efficacy reports without eligible immune outcomes, non-separable mixed populations, and reports superseded by a more complete publication from the same analysis were excluded.

### 2.4 Study selection and data extraction

Records were managed in Rayyan. The original search set contained 247 records after duplicate removal and the updated search identified 39 additional unique records, yielding 286 records for title-and-abstract screening. Of these, 265 were excluded and 21 reports underwent full-text or complete-abstract assessment. The 21 reports all originated from the original Rayyan search set; the 39 updated-search records were excluded at title-and-abstract screening. The registered protocol specified screening by one person with checking by at least one other person. The original screening was performed by one reviewer without the planned second-person check. To reduce classification error, the same reviewer subsequently performed a targeted, temporally separated self-audit of 52 records, comprising all 32 records previously identified as potentially relevant or ambiguous plus a reproducible random sample of 20 records that had appeared clearly excluded. The audit identified no new eligible report after deduplication and full-text reconciliation, and it led to removal of one previously retained orbital-response abstract that lacked an eligible cellular immune-profiling outcome. This procedure was an internal quality-control measure and was not equivalent to independent verification. Full reports were linked by trial registration number, population, sampling time points, and assay to identify overlapping publications. Data extracted included cohort, sample size, compartment, sampling schedule, assay, immune endpoint, clinical association, and publication status.

### 2.5 Evidence units and hierarchy

The primary synthesis unit was the underlying cohort, not the publication. Within each cohort, one principal report was used for each outcome and companion reports contributed only non-duplicated analyses. To aid transparency, reports were assigned descriptive evidence labels: level A, full prospective clinical report with prespecified or clearly described longitudinal sampling; level B, full exploratory translational study or independent prospective biomarker cohort; level C, *post hoc* or small mechanistic cohort; and level D, conference abstract with incomplete methods. These labels were author-developed descriptors, not a validated certainty-of-evidence system and not a substitute for GRADE or formal risk-of-bias assessment. Directness was assessed separately: direct for blood or metastatic-tissue immune measurements and indirect for skin.

### 2.6 Methodological interpretation

A formal domain-level risk-of-bias appraisal was not used as the basis for the final interpretation. Instead, each report was read descriptively with attention to the features most relevant to translational inference: whether the analysis was prospective or exploratory, whether immune sampling was longitudinal, whether samples came from blood, metastatic tumor, or skin, whether the uveal-melanoma population was separable, whether the report was a full article or conference abstract, and whether the data overlapped with other reports from the same trial. These considerations were used to avoid overstating causal or prognostic claims.

### 2.7 Synthesis

Meta-analysis was not performed because reports used overlapping populations, non-equivalent assays and time points, and rarely supplied comparable effect estimates. Reports were grouped by evidence unit and then by mechanistic proposition. Direction and magnitude of within-patient change were summarized when available. The synthesis was descriptive, and statements of confidence were deliberately qualitative, emphasizing consistency, directness, sample size, completeness of reporting, and the number of genuinely independent cohorts.

### 2.8 Deviations from the registered protocol

The final review differed from, or required clarification of, the registered record in six respects. First, the electronic searches preceded PROSPERO submission and registration, and the registered start date of 23 January 2026 does not capture the search conducted on 9 December 2025. Second, the protocol specified that screening, data extraction, and risk-of-bias or quality assessment would each be completed by one person and checked by another; the initial review did not receive this second-person check. A temporally separated self-check reduces error but does not remove this deviation. For this reason, the manuscript is presented as a narrative descriptive review rather than as a completed formal systematic review. Third, a formal risk-of-bias table was replaced by a descriptive account of methodological limitations because most included reports were exploratory companion analyses or abstracts with incomplete domain-level information. Fourth, after full-text assessment and before final synthesis, eligibility was clarified at the outcome level: cytokine, chemokine, and gene-expression findings were synthesized only when reported within an otherwise eligible cellular/tissue-immunology report or evidence unit, whereas stand-alone cytokine-only reports were not retained. Fifth, after full-text assessment, skin was added as a separate indirect compartment because one independent serial skin-biopsy cohort provided human pharmacodynamic evidence, but these data were not treated as direct evidence of metastatic or hepatic tumor remodeling. Sixth, although ctDNA was listed among eligible measurements in PROSPERO, ctDNA-only reports were excluded during full-text reconciliation before final synthesis after clarification that the review question required lymphocyte-subpopulation or cellular immune-profiling outcomes. Finally, reports were consolidated at cohort level after recognition of substantial publication overlap. These clarifications and deviations are reported because each affects interpretation of certainty.

## 3 Evidence architecture and mechanistic themes

### 3.1 Study selection

The original search set contained 247 records after duplicate removal. The updated searches identified 39 additional unique records not present in the original set; all 39 were excluded during title-and-abstract screening. Overall, 286 records were screened, 265 were excluded at the title-and-abstract stage, and 21 reports underwent full-text or complete-abstract assessment (**Figure 1**). Of these, 10 reports were retained and 11 were excluded. Full-text exclusions comprised 3 superseded analyses, 6 reports without an eligible immune/cellular outcome for the formal synthesis, and 2 mixed-population reports in which uveal-melanoma immune findings were not adequately separable. The 10 retained reports should not be interpreted as 10 independent studies; the principal evidence units are summarized in **Table 1**.

**Figure 1:**
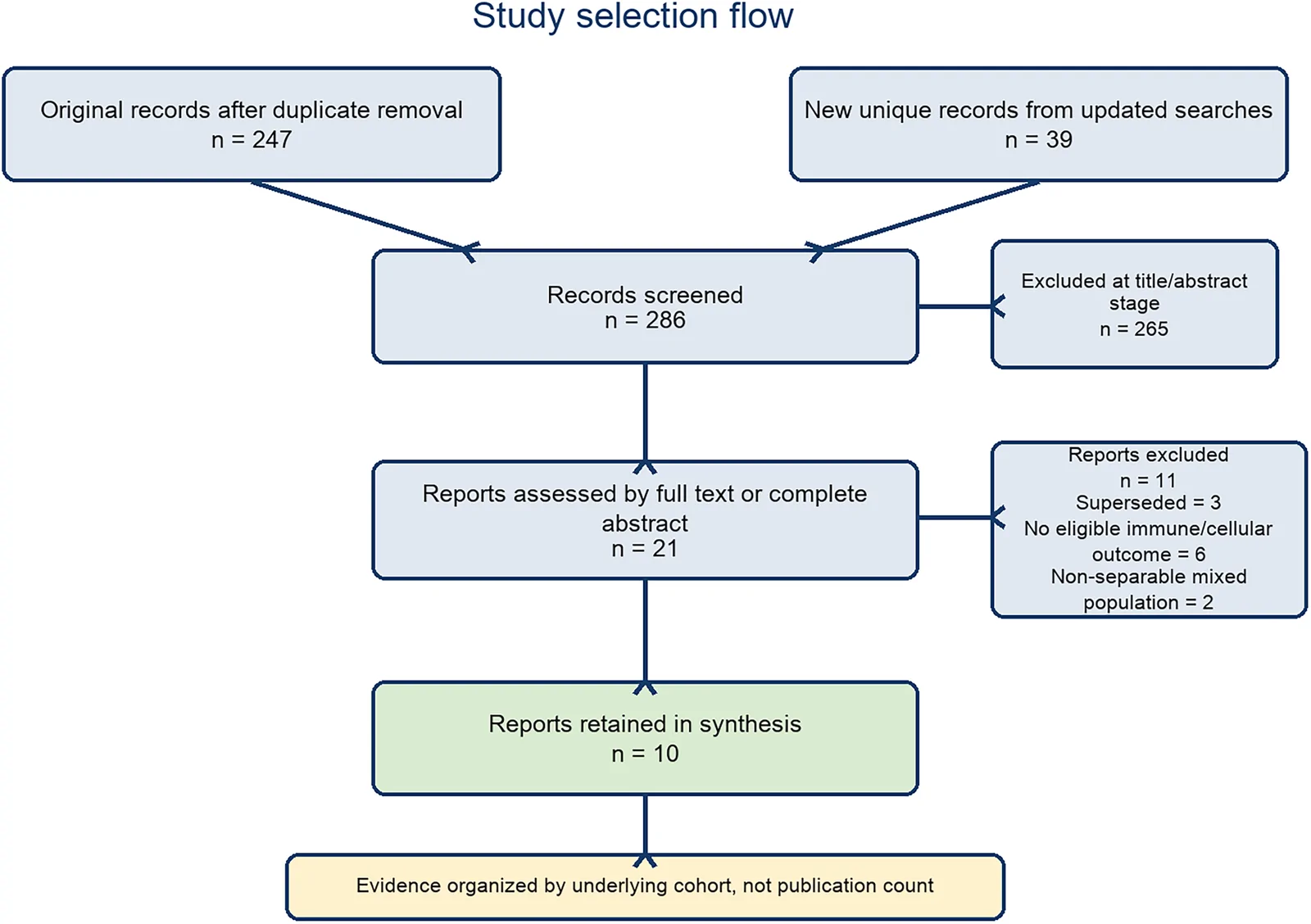
PRISMA-style flow of study selection.

**Table 1:** Principal evidence units.

Evidence unit	Population and design	Retained reports	Primary contribution
NCT02570308	Previously treated mUM; prospective phase 1/2 and phase 2	7	Blood cytokines and lymphocyte redistribution; baseline, day-16, and progression tumor profiling; macrophage and B-cell analyses
NCT03070392	Previously untreated mUM; randomized phase 3	2	Baseline CD163:CD3, tumor infiltration, and serum immune markers
NCT04960891	Independent prospective serial skin-biopsy cohort; n=11	1	Spatial and single-cell cutaneous pharmacodynamics

### 3.2 Architecture of the evidence base

Seven retained reports arose from NCT02570308, 2 from NCT03070392, and 1 from the independent NCT04960891 skin-biopsy cohort. Consequently, the apparent breadth of the literature reflects deep profiling of a few cohorts rather than broad independent replication. Findings from NCT02570308 are therefore described as consistently observed across overlapping analyses from the same trial rather than as independently replicated.

### 3.3 Early systemic activation and cellular redistribution

The most consistent systemic observation was a transient rise in inflammatory and interferon-inducible mediators after dosing. In the early phase 1 cohort, serum cytokine changes were accompanied by a transient reduction in circulating lymphocytes, compatible with redistribution or margination from blood but not proof of trafficking into metastatic lesions (4). In the expanded NCT02570308 analysis, IFN-γ and CXCL9/10/11 peaked approximately 8–24 hours after dosing, alongside IL-2, IL-6, IL-10, TNF-α, and CCL2 (5). A separate pharmacodynamic report associated increased serum CXCL10 and reduced peripheral CXCR3+ CD8+ T cells with longer overall survival (6). Thus, early systemic activation is consistently observed within NCT02570308, whereas its prognostic specificity remains uncertain.

### 3.4 Recruitment and early remodeling in metastatic tissue

Paired biopsies provide direct temporal evidence of treatment-associated changes in metastatic tissue, but they do not prove that the circulating cells measured in blood migrated into those lesions. The early phase 1 report described increased CD3, CD4, and CD8 cells at cycle 1 day 16 in up to 6 paired patient biopsy observations (4). A larger companion analysis reported approximately threefold higher CD3 and twofold higher CD8 density after 3 doses, with induction of interferon-response, antigen-presentation, granzyme B, CXCL9/10/11, IDO1, and PD-L1 transcripts (5). The complete 2025 tumor-landscape report confirmed 2.9-fold and 2.3-fold median increases in CD3 and CD8 cells, respectively, in 57 CD3-evaluable and 56 CD8-evaluable paired tumor observations and reported increased T cell density even in tumors categorized as immune deserted or excluded at baseline (7). Increased T cell density supports treatment-associated tissue change, but by itself does not establish cellular recruitment, functional remodeling, or mediation of survival. Bulk transcriptomic changes may reflect altered cellular composition, altered cell states, or both. These analyses support early metastatic-tissue pharmacodynamic changes, but they are nested within the same trial and therefore constitute 1 principal evidence unit.

Additional NCT02570308 analyses suggested broader treatment-associated immune changes. B cells increased after 3 doses and small lymphoid aggregates became more frequent (8). Macrophage-focused patient-sample analyses showed that a high baseline CD163:CD3 ratio was associated with shorter survival and that on-treatment macrophage transcription shifted toward inflammatory programs (9). The same report also included experimental macrophage models; these model-derived findings are discussed as mechanistic context and were not treated as direct patient-sample evidence. A T cell fitness analysis reported larger increases in tumor CD3 infiltration and inflammatory gene expression among patients with a higher baseline blood fitness signature (10). These findings are biologically coherent but exploratory, partially overlapping, and not adjusted uniformly for multiplicity.

### 3.5 Baseline features and durability of remodeling

Baseline immune-cell abundance alone was not a reliable universal predictor. In the phase 3 biomarker analysis, individual CD3, CD8, or CD163 densities were not associated with improved survival, whereas a lower CD163:CD3 ratio was associated with longer survival in the tebentafusp arm (11). Another phase 3 companion report associated lower baseline CD163:CD3 and lower serum IL-6, IL-10, CXCL10, CXCL11, and MCP1/CCL2 with survival of at least three years (12). MCP1 and CCL2 refer to the same chemokine. These analyses support the relevance of relative myeloid-to-lymphoid balance but do not establish a predictive biomarker because cut points were exploratory and the treatment-by-biomarker interaction was not established.

In NCT02570308, higher baseline expression of interferon-related genes was associated with tumor reduction and survival. Importantly, the magnitude of early recruitment at day 16 was less clearly related to outcome than persistence of antigen-presentation and T cell programs in optional progression biopsies (7). This suggests that durable remodeling may be more clinically relevant than the acute pharmacodynamic peak, but progression-biopsy analyses were small and vulnerable to selection and guarantee-time biases.

### 3.6 Skin as an indirect pharmacodynamic model

An independent cohort of 11 patients underwent serial skin biopsies (13). Acute cutaneous events were accompanied by CD4 and CD8 infiltration, melanocyte loss, T cell proliferation, interferon and cytotoxic programs, and spatial proximity of CD8 cells to melanocytes. These data provide direct evidence of on-target tissue pharmacodynamics in humans and are valuable because they arise outside the major tumor-biopsy trial. Nevertheless, skin differs from hepatic metastases in target-cell context, stromal composition, and immune regulation; the results should not be presented as independent validation of liver-tumor remodeling.

### 3.7 Methodological caveats of the evidence base

The full articles were consistently limited by optional or small biospecimen subsets, exploratory analyses, and incomplete control of multiplicity. Conference abstracts frequently provided insufficient information on participant selection, missing samples, assay methods, confounder adjustment, and selective reporting. These caveats, summarized in **Table 2**, refer to the specific translational analyses rather than to the quality of the parent clinical trials. They are reported descriptively and should not be read as a formal domain-level risk-of-bias assessment.

**Table 2:** Methodological and reporting caveats of retained reports.

Report	Source	Principal caveats
Sacco 2025 (7)	Full article	Optional serial biopsies; exploratory multi-omic analyses; small progression subset
Benlahrech 2025 (10)	Conference abstract	Incomplete selection and assay detail; exploratory subgroup; limited reporting
Güç 2025 (9)	Full article	Nested exploratory subsets; small scRNA-seq sample; extensive mechanistic analyses
Sato 2024 (8)	Conference abstract	Incomplete methods; attrition across time points; negative findings not assessable
Carvajal 2022 (4)	Full article	Paired tumor analyses available in very few patients; exploratory endpoints
Watson 2022 (14)	Conference abstract	Only separable NCT02570308-derived antibody/biopsy/RNA-seq components were analyzed; mixed-melanoma findings were not included when uveal-melanoma-specific data were not separable; multiplicity unclear
Butler 2021 (5)	Conference abstract	Methods and missing samples incompletely reported; multiple biomarkers tested
Orloff 2023 (12)	Conference abstract	*Post hoc* long-survivor definition; multiple biomarkers; incomplete reporting
Piperno-Neumann 2022 (11)	Conference abstract	Baseline-only biomarker analysis; cut-point and interaction details limited
Cutaneous study 2025 (13)	Full article	Small cohort; direct evidence for skin pharmacodynamics but indirect for metastatic or hepatic tumor remodeling

## 4 Discussion

This review changes the interpretation of the tebentafusp translational literature in 2 ways. First, the evidence base is substantially smaller than the publication count suggests. Most cellular and tissue findings derive from repeated analyses of NCT02570308. Second, the proposed systemic-to-tumor axis is supported at the level of temporal coherence but not yet as a complete causal pathway. Cytokines and circulating lymphocyte changes occur within hours, and paired biopsies show increased T cell density and interferon programs by day 16. However, no study directly tracks circulating clones into hepatic lesions or formally tests whether systemic changes mediate survival.

The clearest direct mechanistic conclusion is therefore narrower than previously proposed: tebentafusp is associated with increased T cell density and activation-related transcriptional programs in metastatic tissue, including tumors with low baseline infiltration. The literature also suggests that the resulting microenvironment is not uniformly favorable. PD-L1, IDO1, macrophage programs, and other adaptive or suppressive features are induced alongside cytotoxic and antigen-presentation pathways. The possibility that such programs contribute to limited radiographic response remains hypothesis-generating, because the available studies do not formally test mediation between suppressive programs and imaging outcomes. Target-side determinants, including gp100 expression, peptide-HLA presentation, and HLA class I status, may modify immune recruitment and clinical response and should be incorporated into future paired translational studies.

Skin provides unusually detailed spatial and single-cell evidence of on-target T cell cytotoxicity but remains an imperfect surrogate for the liver microenvironment. Its inclusion as an explicitly indirect compartment adds independent pharmacodynamic evidence without being treated as validation of hepatic-tumor remodeling.

ctDNA-only reports were excluded from the formal cellular immune synthesis because they did not provide eligible lymphocyte-subpopulation or cellular immune-profiling outcomes. Nevertheless, ctDNA remains clinically relevant to the response-survival discordance observed with tebentafusp and should be considered alongside immune pharmacodynamic studies when interpreting early biological response (15). This ctDNA discussion is provided as clinical context and is not part of the formal immune-cell synthesis.

These findings identify a specific research gap and therefore a clearer contribution than a general description of known tebentafusp biology. Prospective studies should collect matched blood and liver biopsies at standardized early and late time points; apply TCR clonotype or cell-state tracking across compartments; prespecify immune endpoints and multiplicity control; and test whether immune changes mediate the relationship between treatment and outcome. Independent validation cohorts are especially important because the current literature is dominated by sponsor-linked analyses of two trials.

## 5 Conclusions

Human studies show that tebentafusp is associated with rapid systemic inflammatory signaling and subsequent increases in T cell density and interferon-associated programs in metastatic tissue. These observations support temporal components of a systemic-to-tumor model, but increased T cell density alone does not prove recruitment, functional remodeling, or mediation of survival, and the complete causal axis remains unproven and has not been independently validated in paired blood and liver samples. Future studies should prioritize independent, longitudinal, multi-compartment validation rather than additional overlapping analyses of the same trial populations.
