# Supplementary material for: Tebentafusp induces systemic immune activation, but tumor immune remodeling remains incompletely validated: a narrative review in metastatic uveal melanoma
Source: Front Immunol. 2026 Oct 12;17:1934256. doi: 10.3389/fimmu.2026.1934256 (PMC13697601; doi:10.3389/fimmu.2026.1934256)
Supplement: Supplementary file 1 [file Table1.docx]

| **Database/platform** | **Updated rerun count (9 Jun 2026)** | **Search strategy originally executed on 9 Dec 2025** |
| --- | --- | --- |
| PubMed/MEDLINE (PubMed) | 45 | (("uveal melanoma"[MeSH Terms] OR "ocular melanoma" OR "choroidal melanoma" OR "uveal melanoma") AND ("tebentafusp" OR "IMCgp100" OR "ImmTAC" OR "gp100 TCR bispecific") AND ("immune infiltrate" OR "tumor microenvironment" OR "lymphocyte subpopulation" OR "T cells" OR "CD8" OR "TIL" OR "immune phenotype" OR "immune biomarkers" OR "ctDNA" OR "cytokines" OR "flow cytometry" OR "scRNAseq")) AND (humans[MeSH Terms]) |
| Embase (Elsevier Embase.com) | 251 | ('uveal melanoma'/exp OR 'uveal melanoma':ti,ab,kw OR 'ocular melanoma':ti,ab,kw OR 'choroidal melanoma':ti,ab,kw) AND ('tebentafusp'/exp OR 'tebentafusp':ti,ab,kw OR 'IMCgp100':ti,ab,kw OR 'ImmTAC':ti,ab,kw OR 'gp100 TCR bispecific':ti,ab,kw) AND ('immune infiltrate'/exp OR 'tumor microenvironment'/exp OR 'lymphocyte subpopulation'/exp OR 'T cells'/exp OR 'CD8'/exp OR 'TIL'/exp OR 'immune phenotype'/exp OR 'immune biomarkers'/exp OR 'ctDNA'/exp OR 'cytokines'/exp OR 'flow cytometry'/exp OR 'scRNAseq'/exp OR 'immune infiltrate':ti,ab,kw OR 'tumor microenvironment':ti,ab,kw OR 'lymphocyte subpopulation':ti,ab,kw OR 'T cells':ti,ab,kw OR 'CD8':ti,ab,kw OR 'TIL':ti,ab,kw OR 'immune phenotype':ti,ab,kw OR 'immune biomarkers':ti,ab,kw OR 'ctDNA':ti,ab,kw OR 'cytokines':ti,ab,kw OR 'flow cytometry':ti,ab,kw OR 'scRNAseq':ti,ab,kw) AND [humans]/lim |
| CENTRAL (Cochrane Library) | 29 | ("uveal melanoma" OR "ocular melanoma" OR "choroidal melanoma") AND (tebentafusp OR IMCgp100 OR ImmTAC OR "gp100 TCR bispecific") AND ("immune infiltrate" OR "tumor microenvironment" OR "lymphocyte subpopulation" OR "T cells" OR CD8 OR TIL OR "immune phenotype" OR "immune biomarkers" OR ctDNA OR cytokines OR "flow cytometry" OR scRNAseq) |

Supplementary Table S1. Electronic search strategies and updated rerun yields. The search strings reproduce the strategies originally executed on 9 December 2025. The database-specific counts shown in the table were obtained when the searches were rerun on 9 June 2026 and should not be interpreted as the original December 2025 per-database yields. The original per-database December 2025 counts are not available; the original deduplicated search set contained 247 records. Platforms used were PubMed for MEDLINE, Elsevier Embase.com for Embase, and the Cochrane Library for CENTRAL.

**Supplementary Table S2. Full-text and complete-abstract eligibility decisions.**

This table reconciles the 21 reports assessed after title-and-abstract screening with the 10 retained reports and 11 full-text exclusions used in the PRISMA-style flow diagram.

| **No.** | **Status** | **Evidence family** | **Report** | **Rationale** |
| --- | --- | --- | --- | --- |
| 1 | Included | NCT02570308 | 2025. Evolution of the tumor immune landscape during treatment with tebentafusp, a T cell receptor-CD3 bispecific | Principal and most complete tumor-biopsy report: baseline, day-16, and progression immune landscape. |
| 2 | Included | NCT02570308 | 2025. TEBENTAFUSP CONTROLS DISEASE AND PROLONGS SURVIVAL BY INCREASING THE T CELL: TUMOR CELL RATIO, ACTIVATING T CELLS AND INHIBITING TUMOR PROLIFERATION IN METASTATIC UVEAL MELANOMA PATIENTS | Eligible paired-biopsy T-cell analysis with a blood T-cell-fitness subgroup; not an independent cohort. |
| 3 | Included | NCT04960891 | 2025. Tebentafusp elicits on-target cutaneous immune responses driven by cytotoxic T cells in uveal melanoma patients | Included as an explicitly indirect skin pharmacodynamic domain. The cohort provides direct evidence of cutaneous pharmacodynamics and is not used as direct evidence of liver or metastatic tumor remodeling. |
| 4 | Included | NCT02570308 | 2025. Tebentafusp, a T cell engager, promotes macrophage reprogramming and in combination with IL-2 overcomes macrophage immunosuppression in cancer | Eligible clinical baseline CD163:CD3 and paired on-treatment macrophage-signature data; preclinical experiments must be synthesized separately. |
| 5 | Included | NCT02570308 | 2024. Development of lymphoid aggregates in metastatic uveal melanoma tumors treated with tebentafusp | Provides on-treatment B-cell and lymphoid-aggregate results not fully reported in the 2025 full tumor-landscape article. |
| 6 | Excluded | NCT02866149 | 2024. Prospective assessment of circulating tumor DNA in patients with metastatic uveal melanoma treated with tebentafusp | Longitudinal ctDNA biomarker study without the review-required lymphocyte-subpopulation or cellular immune-profiling outcome. |
| 7 | Excluded | NCT03070392 | 2023. Early ctDNA reduction is associated with better overall survival in the Ph 3 trial of tebentafusp in previously untreated metastatic uveal melanoma | Phase-3 ctDNA response analysis without the review-required lymphocyte-subpopulation or cellular immune-profiling outcome. |
| 8 | Excluded | NCT03070392 | 2023. Early ctDNA reduction may identify patients with stable disease and long OS on tebentafusp | Restricted ctDNA subgroup analysis without the review-required lymphocyte-subpopulation or cellular immune-profiling outcome. |
| 9 | Excluded | Pooled NCT01211262/NCT02570308/NCT03070392 | 2023. Evidence of tumor response in orbital lesions treated with tebentafusp in metastatic uveal melanoma patients | Excluded after targeted same-reviewer self-audit: orbital-response report without an eligible lymphocyte-subpopulation or cellular immune-profiling outcome for synthesis. |
| 10 | Included | NCT03070392 | 2023. MOLECULAR FEATURES ASSOCIATED WITH LONG SURVIVAL ON TEBENTAFUSP IN PREVIOUSLY UNTREATED METASTATIC UVEAL MELANOMA IN A PHASE 3 TRIAL | Eligible phase-3 baseline tissue and serum biomarker analysis; not an independent study. |
| 11 | Excluded | NCT02570308 | 2022. Clinical and molecular response to tebentafusp in previously treated patients with metastatic uveal melanoma: a phase 2 trial | Phase-2 clinical and ctDNA report without lymphocyte-subpopulation dynamics or an eligible cellular immune-profiling outcome. |
| 12 | Included | NCT03070392 | 2022. Low baseline macrophage to T cell ratio is predictive of overall survival with tebentafusp in a phase 3 trial in metastatic uveal melanoma | Eligible phase-3 validation of baseline CD163:CD3 association; not superseded by the phase-2 macrophage article. |
| 13 | Excluded | NCT02570308 | 2022. MOLECULAR FEATURES IN TUMORS AT TIME OF PROGRESSION ON TEBENTAFUSP ASSOCIATED WITH OVERALL SURVIVAL (OS) | Progression-biopsy analysis superseded by the complete 2025 Cell Reports Medicine tumor-landscape article by Sacco et al. |
| 14 | Included | NCT02570308 | 2022. Phase i Study of Safety, Tolerability, and Efficacy of Tebentafusp Using a Step-Up Dosing Regimen and Expansion in Patients with Metastatic Uveal Melanoma | Eligible systemic cytokine, blood-lymphocyte, and paired-biopsy pharmacodynamic data; earlier 42-patient subset of the same trial. |
| 15 | Included | NCT01211262 + NCT02570308 | 2022. TEBENTAFUSP INDUCED T AND B CELL EPITOPE SPREAD IN PATIENTS WITH ADVANCED MELANOMA | Included only for separable NCT02570308-derived antibody, biopsy, and RNA-seq findings; mixed NCT01211262 TCR-seq findings were not included because the uveal subgroup was not separable. |
| 16 | Excluded | NCT02570308 | 2021. 1770P Genomic correlates of clinical outcomes in patients with metastatic uveal melanoma (mUM) treated with tebentafusp (tebe) | Baseline and on-treatment genomic analyses from the same phase-2 cohort are incorporated into the complete 2025 Cell Reports Medicine tumor-landscape article by Sacco et al. |
| 17 | Excluded | NCT03070392 | 2021. Overall survival benefit with tebentafusp in metastatic uveal melanoma | Pivotal efficacy and safety trial without the protocol-required immune biomarker assessments. Cite as clinical context only. |
| 18 | Excluded | NCT02570308 + NCT03070392 | 2021. Overall survival on tebentafusp in metastatic uveal melanoma (MUM) across the range of tumor GP100 expression levels | gp100-stratified infiltration and activation findings are incorporated in the complete 2025 tumor-landscape article. |
| 19 | Included | NCT02570308 | 2021. Tebentafusp induces transient systemic inflammation and modifies the micro-environment to sensitize uveal melanoma tumors to cytotoxic CD8 cells | Eligible large phase-2 serum cytokine and paired-biopsy immune analysis; overlaps with but is not fully replaced by the early phase-I report. |
| 20 | Excluded | NCT01211262 | 2020. Tebentafusp, a TCR/Anti-CD3 bispecific fusion protein targeting gp100, potently activated antitumor immune responses in patients with metastatic melanoma | Immune analyses combine cutaneous and uveal melanoma; only one uveal patient contributed to the paired-biopsy analysis and results are not adequately separable. |
| 21 | Excluded | NCT01211262 | 2019. Pharmacodynamic effect of tebentafusp (TCR-CD3 bispecific) on peripheral cytokines and association with overall survival in patients with advanced melanoma [6] | Correct source verified as a Melanoma Bridge/Immunotherapy Bridge 2019 abstract from NCT01211262. Excluded because the report describes an advanced melanoma mixed population and does not provide separable uveal-melanoma-specific cellular immune results for synthesis. |

**Supplementary Table S3. Report-level evidence characteristics for retained reports.**

Evidence levels are the descriptive categories defined in the Methods and are not formal GRADE or risk-of-bias ratings. NR indicates not fully reported in the abstract or available report.

| **Report** | **Evidence unit** | **Evaluable sample size** | **Compartment** | **Time points** | **Assay** | **Main quantitative finding** | **Publication status** | **Overlap** | **Sponsorship/relationships** | **Level** |
| --- | --- | --- | --- | --- | --- | --- | --- | --- | --- | --- |
| Sacco 2025 | NCT02570308 | Paired tumor observations: CD3-evaluable n=57; CD8-evaluable n=56 | Metastatic tumor | Baseline, day 16, progression | IHC, RNA-seq/multi-omic profiling | Median CD3 and CD8 density increased 2.9-fold and 2.3-fold, respectively | Full article | Principal tumor-landscape report; overlaps with NCT02570308 companions | Sponsor-linked / Immunocore-affiliated authors | B |
| Benlahrech 2025 | NCT02570308 | NR in abstract | Blood and metastatic tumor | Baseline and on-treatment | Blood T cell fitness signature, biopsy/RNA-seq | Higher baseline blood T cell fitness associated with larger on-treatment tumor immune changes | Conference abstract | Companion analysis; not independent | Sponsor-linked / Immunocore-affiliated authors | D |
| Staeger 2025 | NCT04960891 | n=11 | Skin | Serial skin biopsies | Spatial and single-cell immune profiling | Cutaneous reactions associated with CD4/CD8 infiltration, melanocyte loss, proliferation, and cytotoxic programs | Full article | Independent skin pharmacodynamic cohort; indirect for metastases | Includes industry-affiliated coauthors; independent clinical cohort | B |
| Güç 2025 | NCT02570308 | Nested translational subsets; per-assay n varies | Metastatic tumor and experimental macrophage models | Baseline and on-treatment | scRNA-seq, macrophage signatures, functional assays | Patient samples: high baseline CD163:CD3 associated with shorter survival; on-treatment macrophage programs shifted toward inflammatory states. Experimental macrophage models reported separately as mechanistic context. | Full article | Companion macrophage-focused report; overlaps with NCT02570308 | Sponsor-linked / Immunocore-affiliated authors | B |
| Sato 2024 | NCT02570308 | NR in abstract | Metastatic tumor | After 3 doses | B cell / lymphoid aggregate assessment | B cells and small lymphoid aggregates increased on treatment | Conference abstract | Companion abstract; not independent | Sponsor-linked / Immunocore-affiliated authors | D |
| Orloff 2023 | NCT03070392 | NR in abstract | Tumor and serum | Baseline | Biomarker subgroup analysis | Lower CD163:CD3 and selected serum inflammatory markers associated with long survival | Conference abstract | Phase 3 companion biomarker analysis; not independent | Sponsor-linked / Immunocore-affiliated authors | D |
| Piperno-Neumann 2022 | NCT03070392 | NR in abstract | Tumor | Baseline | IHC / CD163:CD3 ratio | Lower baseline macrophage-to-T cell ratio associated with longer overall survival in tebentafusp arm | Conference abstract | Phase 3 biomarker analysis; predictive interaction not established | Sponsor-linked / Immunocore-affiliated authors | D |
| Carvajal 2022 | NCT02570308 | Patients/specimens: serum n=41-42; blood lymphocyte observations n=32; paired biopsy observations up to n=6 | Blood and metastatic tumor | C1D1, 8-24 h, C1D15/C1D16 | Serum cytokines, lymphocyte counts, IHC | Serum cytokines rose 8-24 h after dosing; paired biopsies showed increased CD3/CD4/CD8 cells in up to 6 patients | Full article | Early subset of NCT02570308; overlaps with later reports | Sponsor-linked / Immunocore-affiliated authors | A |
| Watson 2022 | NCT02570308-derived components only | NR in abstract | Blood and metastatic tumor | Baseline and on-treatment | Antibody, biopsy, RNA-seq analyses | Epitope-spread findings retained only when separable and attributable to NCT02570308 uveal melanoma components | Conference abstract | Mixed-population report; only separable NCT02570308 components used | Sponsor-linked / Immunocore-affiliated authors | D |
| Butler 2021 | NCT02570308 | NR in abstract | Serum and metastatic tumor | After 3 doses | Serum cytokines, IHC, RNA-seq | CD3 and CD8 increased approximately 3-fold and 2-fold; IFN signatures and antigen-presentation genes increased | Conference abstract | Companion abstract; overlaps with later full tumor-landscape report | Sponsor-linked / Immunocore-affiliated authors | D |
